# Influences of Green Outdoors *versus* Indoors Environmental Settings on Psychological and Social Outcomes of Controlled Exercise

**DOI:** 10.3390/ijerph13040363

**Published:** 2016-03-25

**Authors:** Mike Rogerson, Valerie F. Gladwell, Daniel J. Gallagher, Jo L. Barton

**Affiliations:** Centre for Sports and Exercise Science, School of Biological Sciences, University of Essex, Wivenhoe Park, Colchester, Essex CO4 3SQ, UK; vglad@essex.ac.uk (V.F.G.); dgalla@essex.ac.uk (D.J.G.); jobarton@essex.ac.uk (J.L.B.)

**Keywords:** green exercise, wellbeing, directed attention, exercise environments, affect, perceived exertion, intention, exercise behaviour, social interaction

## Abstract

This study addressed a methodological gap by comparing psychological and social outcomes of exercise in green outdoors *versus* built indoors settings, whilst rigorously controlling exercise mode and intensity. The hypotheses were that greater improvements or more desirable values for directed attention, mood, perceived exertion, social interaction time, intention for future exercise behaviour and enjoyment would be associated with outdoors compared to indoors exercise. Following a baseline session, paired participants completed two conditions of 15 min of cycling on an ergometer placed outside in a natural environment and inside in a laboratory setting in a randomized, counter-balanced order. At pre- and post-exercise, directed attention was measured with the digit span backwards task, and mood was assessed with the Profile of Mood States. During the exercise session, visual and verbal interactions were recorded by means of experimenter observations. After each exercise session, participants provided self-reports of their enjoyment of the exercise, perceived exertion and intention for future exercise in the same environment. Social interaction time was significantly greater during outdoors exercise *versus* indoors; on average, participants engaged in three minutes more social interaction during exercise outdoors compared to indoors. Social interaction time significantly predicted intention for future exercise in the outdoors condition, but did not in the indoor condition. There was a significant time by condition interaction for directed attention. Scores worsened in the indoors condition, but improved in the outdoors condition. There was no statistically-significant time by condition interaction for mood and no significant difference between conditions for either perceived exertion or intention. Taken together, these findings show that exercise in a natural environment may promote directed attention and social interactions, which may positively influence future exercise intentions.

## 1. Introduction

Physical inactivity costs the UK economy a total of £7.4 billion per year, £0.9 billion of which is via the National Health Service [1,2]. Regular exercise is associated with better physical and mental health [3,4,5]. A growing pool of evidence suggests that compared to other environments, nature-based (”green”) spaces might promote greater health benefits gained from acute bouts of exercise and might better enable mechanisms that foster future adherence to exercise behaviours [6,7,8,9]. Such a combination of physical activity whilst simultaneously being exposed to nature has been termed ”green exercise” [9,10,11]. 

In particular, exercise environment type is suggested to be important to affective and stress-reduction outcomes of acute exercise bouts. Compared to exercise in indoors or outdoors “built” environments (in which buildings are prominent and often dominated by synthetic or man-made materials), green exercise results in significantly greater affective, attentional and physiological improvements [6,7,8,9,12,13,14,15]. During exercise, nature environments best promote restoration of fatigued cognitive directed attention (the effortful cognitive ability to avoid being distracted by competing stimuli [16,17]), which is beneficial to workplace task performance and may be an underpinning mechanism by which acute bouts of nature-based exercise improve affective states [12,18,19,20,21]. 

The environmental setting also influences perceived exertion during exercise (measured by the Rating of Perceived Exertion (RPE) scale [22,23,24]). During self-paced walking exercise outdoors, individuals walk faster and work harder, but report lower perceived exertion compared to indoors treadmill-based walking [7,25,26]. When asked to adhere to a given perceived exertion, individuals exercise at greater speed, heart rate and blood lactate concentration during outdoors exercise than during indoors exercise [27]. However, in many of these studies, whereas indoors exercise is performed on static ergometers, outdoors exercise constitutes physical movement through an environment. Therefore, the respective extents to which influences on perceived exertion originate from environmental characteristics, such as colour [28], or from differences in optic flow are unknown [29]. Manipulation of optic flow, which is defined as ”the expanding flow on the retina caused by moving through an environment that forms the representational basis of egomotion” (estimating motion relative to a rigid or static scene) [29,30,31,32], has previously been shown to influence perceived exertion during cycling exercise [29]. It is proposed that alongside other factors, such as prior experience, optic flow functions as an important cue for an individual’s internal ”performance template” [33], which is used to assess fatigue and exertion in relation to performance expectations. Restrictions of this cue associated with ergometer-based exercise may therefore influence perceived exertion. In the presence of a possible causal pathway linking exercise intensity and perceived exertion to future exercise behaviours via affective responses [26,34,35], environmental settings may provide a mode of manipulation by which adherence to exercise behaviours may be influenced. Indeed, in a comparison of outcomes of outdoors walks *versus* laboratory-based treadmill walks, individuals reported significantly greater intention to engage in future exercise behaviours following outdoors walking [7].

Social experience may provide an additional pathway by which green exercise might promote future exercise behaviours. Nature-based environments facilitate social interactions within communities, and social cohesion can function as a mediator by which green spaces can influence health [36,37,38]. Indeed, social support can indirectly influence exercise through self-efficacy and outcome expectations and should therefore be included in interventions targeting exercise behaviours [39]. Social expectations of exercise participation significantly contribute to the prediction of exercise frequency in different environmental settings [40]. Although previous research has reported a sample of individuals to have greater social expectations of indoors as compared to outdoors exercise, this may have been influenced by the season being winter when the data were collected [40]. Teas *et al*. [8] noted that during group walking exercise, individuals tended to fall into conversation during outdoors walking, but did not do so during an equivalent indoors condition. Differences in social experiences between exercise environments are important; individuals are more successfully persuaded to partake in physical activity by potential social opportunities associated with exercise sessions than by health benefits [41]. Therefore, green exercise might promote future exercise behaviour by facilitating social interaction and increasing enjoyment of participation [40,42].

Of particular relevance to affective and attentional outcomes, green exercise research is yet to transcend a problematic gap between research paradigms. Some research compares outcomes of exercise either in “built” *versus* nature-based environments [18,43,44,45,46] or outcomes of exercise performed indoors *versus* outdoors [7,8,47]. However, a methodological issue with this paradigm is that research of this kind has often not rigorously controlled the exercise component. This lack of rigorous control is problematic, as exercise intensity and duration influence psychological outcomes [6,35], including perceived exertion [48]. An alternative approach uses ergometers within laboratory settings to control the exercise performed and to examine the importance of the visual exercise environment [9,28,49]. However, this methodology lacks ecological validity, in that viewing environmental scenes alone does not provide the full sensory experience of being present in those environments. An example of this methodological gap is the outcome of directed attention. Improvements in directed attention during exercise have been reported to be facilitated by nature-based settings, both via nature *versus* built outdoors comparisons [18,46] and disparately via the laboratory ergometer-based paradigm [12]. However, research is yet to compare this outcome between indoors and green outdoors environmental settings while controlling for duration and level of intensity of the exercise.

The current study sought to address the methodological gap in existing research of this kind by comparing a range of previously-reported psychological outcomes of exercise in green outdoors *versus* indoors settings, whilst rigorously controlling the exercise component. Participants took part in pairs in order to investigate how environmental setting might influence social experiences of exercise. The hypotheses were that: (i) outdoors exercise would facilitate improvements in directed attention from pre- to post-exercise to a greater extent than indoors exercise [12,18,21]; (ii) outdoors exercise would facilitate improvements in mood from pre- to post-exercise to a greater extent than indoors exercise [6,7,8,9]; (iii) perceived exertion would be lower during outdoors exercise compared to indoors exercise [7,25,26,27]; (iv) accumulative time spent socially interacting during the exercise session would be greater during outdoors exercise compared to indoors exercise [8]; (v) greater intention for a future exercise behaviour would be reported following outdoors exercise compared to built indoors exercise [7]; (vi) social interaction time would predict intention for future exercise behaviour [8,39,40,41]; and (vii) greater enjoyment of the exercise session would be reported following outdoors exercise compared to indoors exercise [7]. 

## 2. Materials and Methods

The following methodology was approved by the University of Essex Ethics Committee (10/BS-S/182/MR). The study was conducted in accordance with the Declaration of Helsinki. All participants provided written informed consent for their participation, by their signing of an informed consent document. 

### 2.1. Participants

Twenty-four participants (19 females, 5 males; age range 18–73 years (*M* = 35.1 ± 20.1 years); stature = 166.8 ± 8.0 cm; mass = 70.2 ± 15 kg) were recruited in pairs from staff (1 participant) and student (10 participants) populations at the University of Essex, as well as from the general public in Essex (13 participants). Each pair of participants knew each other prior to their participation. Participants responded to written and verbal advertisements in which they were asked to sign up as a pair, alongside a friend, family member or romantic partner. Self-pairing of participants was used in order to maintain ecological validity; that is, individuals typically exercise with a partner who they know and with whom they choose to exercise. All participants stated that they were familiar with cycle ergometer-based exercise, except for one participant, who stated that she had never previously experienced using cycle ergometers. Beyond this, participants’ exercise backgrounds were not attained.

### 2.2. Design and Procedure

**Figure 1:**
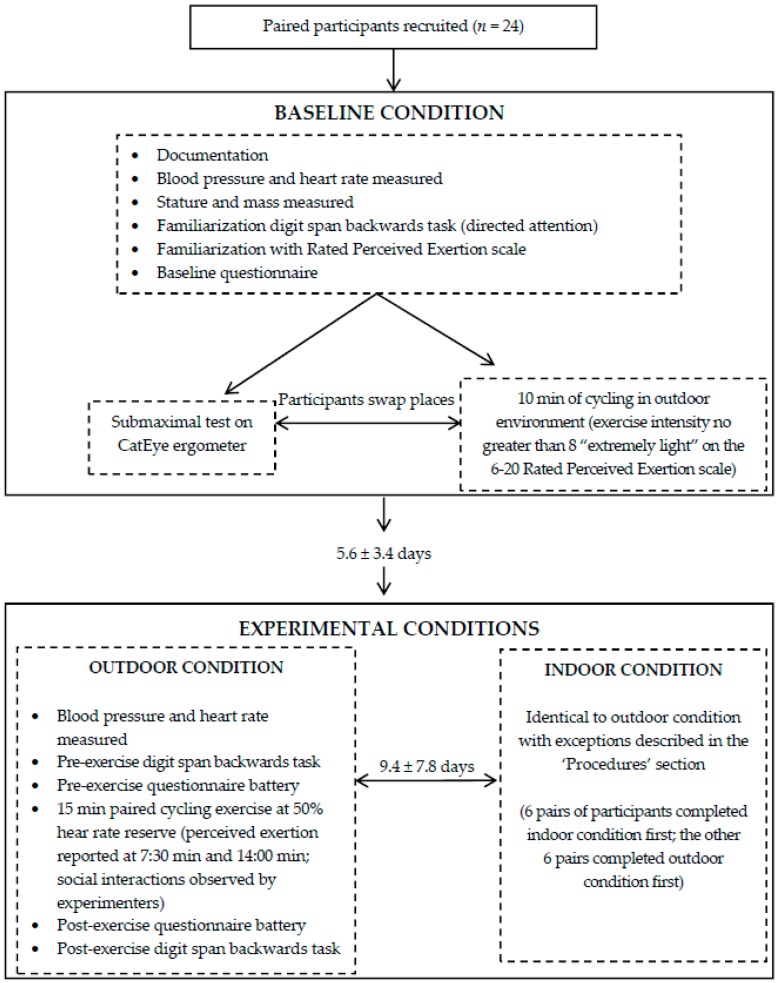
Overview of sessions.

A within-participants design was used, whereby all participants completed each of three conditions: baseline; outdoors; and indoors (Figure 1). For each pair of the participants, conditions were completed on separate days at the same time of day (±1 h). All participants completed the baseline condition first. The mean number of days between experimental occasions was 9.4 ± 7.8 days. The mean number of days between participants’ baseline occasion and first experimental occasion was 5.6 ± 3.4 days.

Paired participants completed experimental conditions (outdoors; indoors) in randomized, counter-balanced order; that is, 12 pairs of participants were randomly assigned to complete the outdoors condition before the indoors condition, with the remaining 12 pairs completing the experimental conditions in the opposite order. The baseline condition was used in order to familiarize participants with each of the experimental measures, equipment and routine and to obtain physiological data that enabled the calculation of each participant’s 50% Heart Rate Reserve (HRR) for use in the experimental conditions. Experimental conditions differed only by the environment in which the exercise of the session was performed. 

### 2.3. Description of Environmental Settings

**Figure 2:**
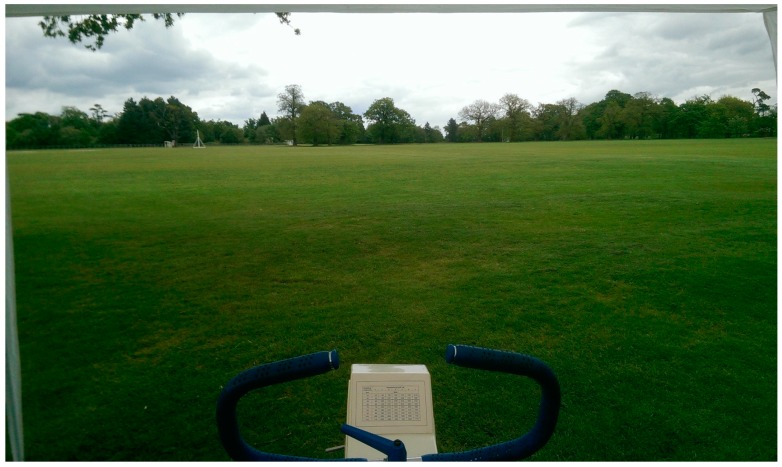
View from the cycle ergometer within the baseline and outdoors conditions.

**Figure 3:**
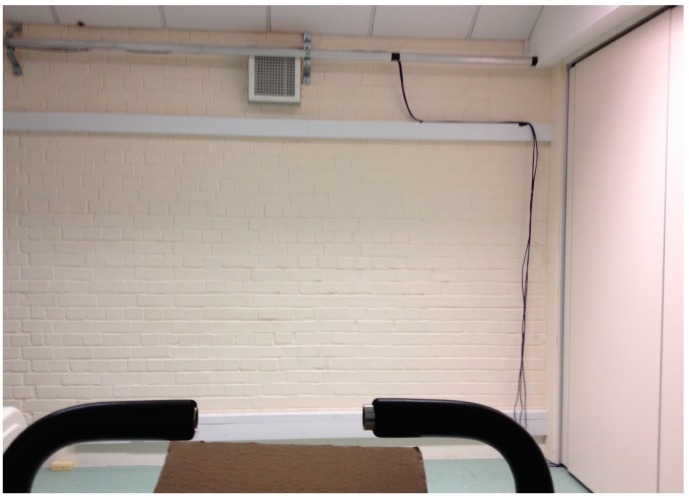
View from the cycle ergometer within the baseline and indoors conditions.

In the outdoors condition, exercise was performed outside, on the University of Essex sports fields, which is a large area of largely level gradient, maintained grassland, lined and partly interspersed with trees (see Figure 2). In the indoor condition, exercise was performed indoors, in a laboratory setting, whereby participants’ view was of a white painted brick wall (Figure 3). The dimensions of the laboratory were 8.3 m × 4.9 m. The experimenters were based at desks placed in one corner of the laboratory. Cycle ergometers were placed approximately in the centre of the laboratory, with participants’ table and chairs positioned between the ergometers and the experimenters’ desks. The laboratory was set out consistently between conditions and between participants.

To ensure consistency between conditions, in both environmental settings, the researchers were based at a desk positioned 2 m diagonally behind the cycle ergometers. It was intended that the sound of the fan/air conditioning unit in the laboratory of the indoors condition would replicate, to some extent, breeze-related sound in the outdoors condition, in an attempt to prevent participants from feeling that their interactions were more observed or listened to in one condition compared to the other.

### 2.4. Equipment

CatEye ergociser (EC-1600, CatEye Co. Ltd., Osaka, Japan) ergometers were used in all conditions. In the baseline condition, an in-built submaximal fitness “test” mode within the ergometer was used (derived from the YMCA bicycle submaximal fitness test [50]). In the experimental conditions, the CatEye ergociser enabled rigorous control of exercise intensity. Intensity was set using the CatEye’s “constant” mode, whereby resistance is continually automatically adjusted in response to fluctuations in pedal cadence in order to maintain constant intensity wattage.

### 2.5. Measures

The pre-exercise questionnaire of the experimental conditions consisted only of the selected measure for mood. Baseline and post-exercise questionnaires were a composite, comprising measures of mood, enjoyment of the exercise session and intention for a future exercise behaviour.

#### 2.5.1. Directed Attention

The digit span backwards task [51] requires attentional effort to mentally hold, track and rearrange items within the short-term memory [18,52]. It has frequently been used as a measure of directed attention [16,18,53,54,55]. The manner and details of the use of the digit span backwards task in the current study was identical to its use by Rogerson and Barton [12].

Participants viewed strings of numbers that were displayed on a computer screen before writing their answers. Numbers were presented serially for a duration of one second each. Rules were given to the participants both verbally by the experimenter and visually on the screen, prior to each test commencing. The instructions were: do not write anything down until prompted to by the screen; when you are writing down your answer, you must physically write in the direction from left to right; do not mouth the numbers at all, at any time; if you cannot remember, please make the best guess that you can. One experimenter was positioned in order to ensure that participants adhered to the given rules. Participants attempted to recite nine number strings, of 3–11 digits in length, which increased in order. Participants scored one mark for each string successfully recited backwards in its entirety. Therefore, the maximum possible score was nine, and the minimum possible score was zero. Four variations of the test were completed by participants in randomised order, before and after the exercise sessions. The familiarization digit span backwards task, which was administered during the baseline condition, comprised four number strings, which progressed from 3–6 digits in length.

#### 2.5.2. Mood

The shortened, 30-item version of the Profile Of Mood States [56,57] was used to measure mood. Participants are required to indicate how they felt “right now” in response to single-word mood descriptor items, along at 5-point Likert-type scale (0, “not at all”, to 4, “extremely”). Five mood descriptor items comprise each of six subscales (anger, confusion, depression, fatigue, tension and vigour). For each subscale, raw scores were converted to *T* scores [57], and an overall mood score (total mood disturbance) was calculated by summing all of the negative mood factors (tension, depression, anger, fatigue, confusion) and subtracting the positive vigour score. Higher total mood disturbance scores indicate poorer overall mood (maximum = 282; minimum = 112). Cronbach’s alpha values of 0.67–0.93 and 0.84–0.95 have been reported, indicating acceptable internal consistency [57,58]. The shortened version of the Profile Of Mood States is suitable for use in exercise contexts [59].

#### 2.5.3. Social Interaction Time

Social interaction time was measured using a stopwatch during the main exercise of each condition. One of the two experimenters was positioned behind the participants and continually visually and audibly observed for visual and verbal interaction between the participants. Social interaction periods were timed accumulatively to give the social interaction time value.

#### 2.5.4. Perceived Exertion

Perceived exertion was measured with the RPE scale [23,24]. The scale comprises a fifteen-point vertical list of numbers, some of which are accompanied by a descriptor word, from 6 “no exertion” to 20 “maximal exertion”. Weighted mean reliability coefficients of physiological measures have been reported for RPE scores: heart rate (0.62); blood lactate concentration (0.57); % VO_2_max (0.64); VO_2_ (0.63) and respiration rate (0.72) [60].

#### 2.5.5. Intention for a Future Exercise Behaviour and Enjoyment of Exercise Session

A 100 mm line was used to measure each of the participants’ enjoyment of the exercise and their intention for a future exercise behaviour. For the measure of enjoyment, participants were instructed to “Put a mark on the line to indicate how much you enjoyed the exercise session”. The continuum ranged from “not at all” (0) to “very much” (100). Similarly, for the measure of intention, participants responded to the question: “Would you attend a free exercise session in the same place that you did your cycling exercise today? Mark the line to indicate your intention”. The continuum ranged from “I definitely will not attend” (0) to “I will definitely attend” (100). For each measure, participants were required to draw a mark somewhere along the respective 100-mm continuum line. Both of these items were within the post-exercise questionnaire only. 

### 2.6. Procedures

Two experimenters were present during all sessions. This enabled simultaneous supervision of participants in different places within the baseline session and simultaneous taking of measurements (e.g., blood pressure, perceived exertion) in the experimental sessions.

#### 2.6.1. Baseline Condition

On arrival at the laboratory, participants sat on a chair at the table whilst the experimenter provided a verbal overview of the content of the study, before participants read a Participant’s Information document and then completed a Physical Activity Readiness Questionnaire (PAR-Q) and informed consent documentation. Participants’ blood pressure was then measured in the seated position, using an Omron MX3 blood pressure monitor (Omron Healthcare UK Ltd., Milton Keynes, UK). Together with the PAR-Q, this served as a screening measure for participants’ suitability for the exercise of the session. Answers on the PAR-Q that suggested exercise-related physiological risk that were not then elaborated on satisfactorily (e.g., reporting experiencing back pain, but elaborating that their doctor had confirmed that they were still safe to perform exercise) would result in exclusion from participation. Participation was only allowed when participants’ blood pressure values were less than 200 (systolic)/100 (diastolic). No participants were excluded from the study. Participants’ stature and mass were then measured. Participants returned to sit at the table and completed a familiarization digit span backwards task (directed attention), before being familiarized with the RPE scale and completing a baseline questionnaire (these measures are not reported here).

Participants were then split in order to complete the remainder of the baseline session separate from their paired participant. One participant remained in the laboratory with one experimenter and was fitted with a heart rate monitor before completing a submaximal fitness test on a CatEye cycle ergometer; the test lasted for 10 min in total, comprising a one-minute rest period and three 3 min exercise stages, whereby resistance is informed by participants’ resting heart rate and heart rate in previous stages. This was completed in order to identify an exercise intensity of 50% HRR, for use in the experimental sessions. Meanwhile, the remaining experimenter walked the remaining participant to the University’s sports fields, where the participant completed 10 min of cycling on a cycle ergometer (see Figure 2). The experimenter instructed this participant to cycle at a RPE intensity of ”8, extremely light”. The purpose of this exercise was to familiarize participants with using the ergometer in the field environment, in order to minimize novelty effects during the experimental sessions. Upon completion, the participants (with experimenters) then swapped and completed the remaining task. Upon completion of the second task, both participants and experimenters assembled in the laboratory, where participants were debriefed, marking the end of the test occasion.

#### 2.6.2. Outdoors Condition

Upon arrival at the laboratory, participants were sat at the table, given a verbal briefing by the experimenter and re-familiarised with the RPE scale, after which blood pressure was taken. Participants then completed the pre-exercise digit span backwards task (directed attention), followed by the pre-exercise questionnaire battery. Experimenters then led the participants to the university sports fields (the same location as used in the baseline condition), where two CatEye cycling ergometers were placed next to each other, one meter apart, with a vista of the sports fields. Upon ensuring that the seat and handlebar heights were comfortable and appropriate, the experimenters instructed participants: “we would now like you to complete 15 min of cycling exercise. The bikes have been programmed for you and we will ask you to report your rated perceived exertion at a couple of points during the exercise. Please report this to us by pointing to the number rather than saying it verbally. Although this is research, please feel free to talk as much or as little as you like”. Participants then completed 15 min of cycling at an intensity of 50% heart rate reserve. At 7:30 min and at 14 min into the exercise, the experimenters simultaneously asked participants to report their perceived exertion using the RPE scale. For this, the experimenters stood on the outer side of the pair of ergometers, so that participants turned away from each other when reporting perceived exertion, so as to avoid awareness of and therefore influence upon each other’s answers.

Upon cessation of exercise, the experimenters led participants back to the laboratory. Participants then sat at the table and completed the post-exercise questionnaire followed by the post-exercise digit span backwards task (directed attention). Participants were debriefed and thanked for their participation.

For health and safety reasons regarding the electricity-powered CatEye cycle ergometers, the outdoor condition was completed only if it was not raining at the time of testing. However, there were no instances of the outdoors condition cancellation due to this reason.

#### 2.6.3. Indoors Condition

The indoors condition was identical to the outdoors condition with a couple of exceptions. Regarding where the exercise of the session was performed, in the indoors condition, the 15 min cycling exercise was performed in the laboratory. Whereas in the outdoors condition, participants walked to and from the sports fields, in the indoors condition, the experimenter led participants on four minutes, entirely indoors walks around the laboratory building and sports centre buildings, both immediately prior to and following the main cycling exercise.

### 2.7. Statistical Analysis

For the measures of social interaction time, intention for a future exercise behaviour, enjoyment of the exercise session and perceived exertion, a series of mixed within (condition: outdoor, indoor)—between (condition order: outdoor first, indoor first) ANOVAs were used to compare values reported in outdoor and indoor conditions. Regressions were also conducted in order to further examine the relationships between social interaction time and the outcome of intention for each environmental condition. For the measures of directed attention and mood, mixed within (condition: outdoor, indoor; time: pre-exercise, post-exercise)-between (condition order: outdoor first, indoor first) repeated measures ANOVA and mixed within-between repeated measures MANOVA were used where appropriate in order to identify potential time by condition interactions. An alpha level of 0.05 was used to indicate statistical significance, with partial eta squared effect sizes (*η_p_*^2^) and 95% confidence intervals (CI) included in order to indicate the magnitude of the effects. 

## 3. Results

For all measures, mean and standard deviation values are reported in Table 1. 

### 3.1. Directed Attention

**Figure 4:**
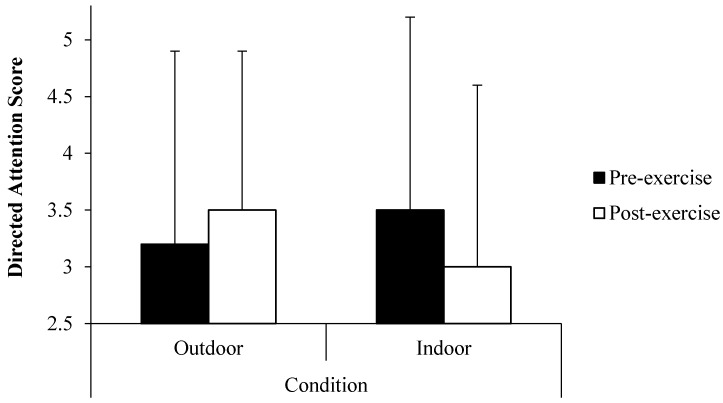
Mean (±1 SD) pre- and post-exercise directed attention scores by condition.

For Hypothesis (i), a paired samples *t*-test showed that outdoors and indoors pre-exercise directed attention did not significantly differ (*t*_23_ = −1.37, 95% CI (−0.73, 0.15), *p* > 0.05). A within (condition: outdoor, indoor; time: pre-exercise, post-exercise)-between (condition order: outdoor first, indoor first) repeated measures ANOVA found that there was a statistically-significant condition × time interaction effect (*F*_1,22_ = 6.21, *p* = 0.02, *η_p_*^2^ = 0.22). Pairwise comparisons showed that whereas directed attention worsened in the indoors condition (*M* ∆ = −0.4, 95% CI for difference (−0.11, 0.94), *p* > 0.05), it improved in the outdoors condition (*M* ∆ = 0.3, 95% CI for difference (−0.01, 0.68), *p* > 0.05; Figure 4). There was no significant condition order × condition × time interaction effect (*p* > 0.05).

### 3.2. Mood

For Hypothesis (ii), a within-between repeated measures MANOVA indicated that there was no statistically-significant time by condition interaction for mood (*F*_6,17_ = 1.70, *p* > 0.05, *η_p_*^2^ = 0.38) and no significant main effect for either condition (*F*_6,17_ = 1.27, *p* > 0.05, *η_p_*^2^ = 0.31) or time (*F*_6,17_ = 2.33, *p* > 0.05, *η_p_*^2^ = 0.45; Table 2). There was no significant condition order × condition × time interaction effect (*F*_6,17_ = 1.02, *p* > 0.05, *η_p_*^2^ = 0.26).

### 3.3. Perceived Exertion, Social Interaction and Intention

A series of within-between ANOVAs found that there were no significant effects for condition for the measures of perceived exertion (Hypothesis (iii)) at 7:30 min and 14:00 min, intention for future exercise behaviour (Hypothesis (v)) and enjoyment of the exercise session (Hypothesis (vii)) (all *p*-values > 0.05). For each of these measures, there were also no significant condition order × condition × time interaction effects (all *p*-values > 0.05).

For the measure of social interaction time (Hypothesis (iv)), a within-between ANOVA found a statistically-significant effect for condition (*F*_1,22_ = 44.79, *p* < 0.001, *η_p_*^2^ = 0.67), whereby social interaction time was greater in the outdoors condition compared to the indoors condition.

For Hypothesis (vi), for the outdoors condition, a simple linear regression found that intention for future exercise behaviour was significantly positively predicted by social interaction time (*F*_1,22_ = 12.23, b = 0.06, *p* < 0.01). For the indoors condition, no statistically-significant equation was found (*p* > 0.05).

## 4. Discussion

The current study aimed to address the methodological gap in existing research of this kind by comparing a range of previously-reported psychological outcomes of exercise performed in green outdoors *versus* indoors environments, whilst controlling the exercise component. In addition to the control of the exercise component, further strengths of the current study were its robust within-subjects, randomised and counter-balanced condition order design; and that its use of a social exercise setting (coupled with the control of the exercise component) transcends the previously-exiting gap in research regarding the influences of exercise environments, which have either controlled the exercise component or used a social setting.

Hypothesis (i) that the outdoors condition would best promote improvements in directed attention was supported. This is consistent with both attention restoration theory [18,20,61] and previous findings supporting the suggestion that compared to “built” indoors or outdoors environments, nature environments facilitate attention restoration during medium intensity exercise [12,18]. Whereas previous studies reported such findings in relation to walking exercise whereby individuals exercised alone, the current study employed cycling exercise using ergometers and examined individuals exercising in pairs. Thus, considered together with previous research, the results allude to the idea that attention restoration can be facilitated by environmental settings during exercise of different modes, in different social settings and without actual or simulated movement through the environment. That directed attention worsened in the indoors condition is in contrast to previous research and fails to support the suggestion that exercise, in general, promotes directed attention improvements [12,62,63,64]. However, this finding should be considered with caution, as although the difference in pre-exercise directed attention was not statistically significant, the direction of the difference in these values did contribute to the reported significant time by condition interaction for this measure. Indeed, there was less scope for improvement following the pre-exercise digit span backwards task in the indoors condition. 

The results failed to support Hypotheses (v) (intention); (ii) (mood) and (vii) (enjoyment), that participants would report greater values for these measures in the outdoors compared to the indoors condition. These findings are in contrast to previous research of this kind [6,7,8,9,14], but considered together, are not inconsistent with the suggested pathway linking exercise intensity and perceived exertion to future exercise behaviours via affective responses [26,34,35]. As enjoyment is a tertiary emotion [65,66], the lack of significant difference in the measure of enjoyment between conditions was consistent with the lack of time by condition interaction for the measure of mood. This combination of results is in line with previous research, which reported the outcome of mood to correlate with, and be predicted by, enjoyment of participation in a bout of green exercise [7,11]. When the exercise performed is comparable, environmental settings appear not to directly influence intentions for future exercise. As exercise intensity and duration influence psychological outcomes of exercise [6,35,48], a possible explanation for the results’ unexpected lack of support for Hypotheses (v) (intention), (ii) (mood) and (vii) (enjoyment) is that in previous research, outdoors *versus* indoors differences in the exercise performed may have contributed to the reported outcome differences [7,25,26,27]. For example, whereas the current study controlled exercise intensity, Focht [7] reported participants’ self-selected intensities of 57% and 59% HRR indoors and outdoors, respectively, although this difference was not accounted for in the analysis of the measure of intention. The theoretical and practical implication of these findings is that differences in physiological qualities of exercise associated with different environmental settings may be the greatest contributor to previously-reported, practically-attainable environment-related differences in affective outcomes of exercise.

The lack of a significant main effect for time on mood was unexpected given that exercise per se is frequently suggested to improve affective state [67,68]. Dual-mode theory proposes that the interplay between and shifting relative importance of collections of cognitive (e.g., self-efficacy, self-presentational concerns) and physiological elements (e.g., acidosis, core temperature) of exercise experiences influences the exercise-affect relationship [69]. It is plausible that the context (*i.e.*, social setting, in a research context) and settings (e.g., of a duration great enough to promote fatigue in some participants) of the exercise performed in the current study may have predisposed characteristics of and the relative importance of particular cognitive and physiological elements; which may in turn not have been conducive to promoting a positive exercise-affect relationship. To speculate, aside from a possible pathway involving attention restoration, as the social setting of the exercise in the current study was different from that of previous research, affect associated with social interactions during the exercise might have functioned to distract from or over-ride other pathways via which both exercise and environments influence affect. However, the data generated by the current study do not enable investigation of this speculation. Indeed, as previous research of this kind has most frequently focussed on affective outcomes of individuals exercising alone, a theoretical and practical implication of the current findings is that potential enhancement of affective exercise outcomes via environment settings may not be generalizable across different social settings. 

Considering the findings for Hypothesis (i) (directed attention), together with the findings for Hypothesis (ii) (mood), fails to support the suggestion that attention restoration theory might partly underpin previously-reported affective benefits of green exercise; that is, that positive affective valence may occur as a side effect of attention restoration [12,18,19,20,21]. Phenomenological and ecological dynamics approaches offer alternative methodologies for elucidating the underpinning mechanisms of previously-reported affective outcomes of green exercise; that is, examination of how characteristics of nature environments might afford greater opportunities for affect-influencing individual-environment interactions than do characteristics of indoors environments; and understanding the individual’s lived experience of these interactions [70,71,72].

Hypothesis (iv), that accumulative time spent socially interacting during the exercise session would be greater during outdoors exercise compared to indoors exercise, was supported. This is consistent with the findings of Teas *et al.* [8]. Although the results did not directly support Hypothesis (v) (intention), Hypothesis (vi) (social interaction time predicting intention) was partially supported. As intention can in part influence behaviour [73,74,75], the finding that intention for future exercise behaviour was positively predicted by social interaction time in the outdoors condition, but not in the indoors condition, both suggests that green exercise settings may facilitate more meaningful social interaction during exercise and supports the notion that environmental exercise settings might influence behavioural choices via a pathway involving social experience [39,40]. A practical implication of this finding is that for exercise contexts in which social experience is deemed important, the environment in which the exercise is performed should be considered. However, future study of and potential future application of an exercise environment-social experience-intentions-behaviour pathway should incorporate consideration of a range of other conceptual (e.g., existing habits of an individual) and measure-related factors that influence the intention-behaviour relationship [76].

The results did not support Hypothesis (iii), that perceived exertion would be lower during outdoors exercise compared to indoors exercise. This is inconsistent with previous research making such comparisons [7,25,26,27]. Although it is plausible that this result occurred because participants effectively expended more perceived effort socially interacting during outdoors exercise (therefore offsetting against differences in perceived exertion that might otherwise have been reported), this possibility cannot be elucidated within the current data, and the current results support the assertion that optic flow at least partially underpins the influences of the environment on perceived exertion during exercise [29]. 

It was beyond the scope of the current research to relate reported exercise intention to actual future exercise behaviours. Future research would benefit from longitudinal designs in this way. In order to better inform designs of therapeutic applications of green exercise, future research should also consider designs that enable relationships and possible interactions between environment and social exercise settings to be elucidated and the roles of exercise mode and social settings to be examined. Such research would clarify remaining caveats, such as: whether reported environmental influences occur when exercise groups are greater in number; when exercise mode is different from those already investigated, for example resistance exercise; or when required focus on the exercise component is greater, for example during games or sports. 

### Limitations

In the baseline condition, half of the participants completed the submaximal cycling test shortly after 10 min of “extremely light” intensity cycling outside, and this may have biased the results of the submaximal test. That is, particularly for participants who exercise less frequently, even “extremely light” intensity exercise may affect the heart rate response of a subsequent bout, thereby biasing the estimated 50% HRR intensity levels used for the experimental conditions. To help guard against this potential bias, resting heart rate values were obtained at a prior stage of the baseline condition session, and participants undertook a two-minute rest period immediately prior to completing the submaximal test.

A further limitation of the current study was that neither participants’ exercise background, nor their relationship with their exercise partner was controlled for. It is possible that some participants were more experienced and comfortable cycling at 50% HRR intensity than other participants. Additionally, day of the week was not consistent between test occasions and was not controlled for within analyses of the data. This was a limitation of the study, as day of the week has been shown to influence affective state [77].

It should be noted that where the current study compared a laboratory to a “green”, nature-based outdoors environment, this represents only one specific environmental comparison. Although the findings have been discussed in relation to previous research of this kind, the current results may have been different if the environments compared were different examples of “green” outdoors or laboratory environments. This denotes a need for further research to examine alternative examples of indoors and outdoors environments, such as gymnasium (indoors) and forest or beach environments (outdoors).

## 5. Conclusions

Restoration of directed attention can be facilitated by environmental settings during exercise of different modes, in different social settings and without actual or simulated movement through the environment. However, the results fail to support the suggestion that attention restoration theory may partly explain previously-reported affective benefits of green exercise. The findings of the current study allude to the idea that previously-reported influences of environmental exercise settings on affective exercise outcomes may have been significantly contributed to by differences in the exercise performed. However, environmental settings might influence behavioural choices via a pathway involving social experience. The marriage of current quantitative methods with phenomenological and ecological dynamics approaches may aid in the understanding of how and why environmental settings of exercise might influence selected outcomes.

## Figures and Tables

**Table 1:** Mean pre- and post-exercise (±1 SD) values by condition.

Measure	Pre-Exercise Values by Condition		Post-Exercise Values by Condition	
	Outdoors	Indoors	Outdoors	Indoors
Intention for future exercise behaviour			68.3 ± 22.0	69.7 ± 25.4
Overall mood	148.8 ± 20.6	145.9 ± 15.1	145.5 ± 16.3	142.2 ± 14.0
Enjoyment of exercise session			65.9 ± 23.5	64.2 ± 23.4
Perceived exertion at 7:30 min			11.9 ± 2.5	12.2 ± 2.5
Perceived exertion at 14:00 min			12.9 ± 2.2	13.5 ± 2.2
Social interaction time (seconds)			613.6 ± 222.2	415.8 ± 263.9
Directed attention (number of strings successfully recited backwards)	3.2 ± 1.7	3.5 ± 1.4	3.5 ± 1.7	3.0 ± 1.6

**Table 2:** Mean pre- and post-exercise (±1 SD) mood subscale values by condition.

Mood Subscale	Pre-Exercise Values by Condition		Post-Exercise Values by Condition	
	Outdoors	Indoors	Outdoors	Indoors
Tension	32.6 ± 2.9	32.5 ± 2.0	32.4 ± 2.1	32.2 ± 1.6
Depression	37.5 ± 1.1	37.6 ± 1.1	37.3 ± 0.8	37.2 ± 0.5
Anger	38.0 ± 1.8	38.0 ± 1.4	37.4 ± 1.7	37.3 ± 1.0
Vigour	35.9 ± 8.6	37.1 ± 7.2	37.2 ± 8.4	38.6 ± 8.6
Fatigue	40.0 ± 7.9	38.8 ± 6.6	39.3 ± 5.1	39.4 ± 4.7
Confusion	36.5 ± 4.1	36.1 ± 3.5	36.3 ± 3.5	34.6 ± 2.9
